# State-of-the-Art on the Sulfate Radical-Advanced Oxidation Coupled with Nanomaterials: Biological and Environmental Applications

**DOI:** 10.3390/jfb13040227

**Published:** 2022-11-07

**Authors:** Sijia Li, Manlin Qi, Qijing Yang, Fangyu Shi, Chengyu Liu, Juanrui Du, Yue Sun, Chunyan Li, Biao Dong

**Affiliations:** 1 Department of Prosthodontics, Jilin Provincial Key Laboratory of Tooth Development and Bone Remodeling, School and Hospital of Stomatology, Jilin University, Changchun 130021, China; 2 Department of Oral Implantology, School and Hospital of Stomatology, Jilin University, Changchun 130021, China; 3 State Key Laboratory on Integrated Optoelectronics, College of Electronic Science and Engineering, Jilin University, Changchun 130012, China

**Keywords:** sulfate radicals, nanomaterials, antimicrobial, environmental, sulfate radical-based advanced oxidation processes (SR-AOPs)

## Abstract

Sulfate radicals (SO_4_^−^·) play important biological roles in biomedical and environmental engineering, such as antimicrobial, antitumor, and disinfection. Compared with other common free radicals, it has the advantages of a longer half-life and higher oxidation potential, which could bring unexpected effects. These properties have prompted researchers to make great contributions to biology and environmental engineering by exploiting their properties. Peroxymonosulfate (PMS) and peroxydisulfate (PDS) are the main raw materials for SO_4_^−^· formation. Due to the remarkable progress in nanotechnology, a large number of nanomaterials have been explored that can efficiently activate PMS/PDS, which have been used to generate SO_4_^−^· for biological applications. Based on the superior properties and application potential of SO_4_^−^·, it is of great significance to review its chemical mechanism, biological effect, and application field. Therefore, in this review, we summarize the latest design of nanomaterials that can effectually activate PMS/PDS to create SO_4_^−^·, including metal-based nanomaterials, metal-free nanomaterials, and nanocomposites. Furthermore, we discuss the underlying mechanism of the activation of PMS/PDS using these nanomaterials and the application of SO_4_^−^· in the fields of environmental remediation and biomedicine, liberating the application potential of SO_4_^−^·. Finally, this review provides the existing problems and prospects of nanomaterials being used to generate SO_4_^−^· in the future, providing new ideas and possibilities for the development of biomedicine and environmental remediation.

## 1. Introduction

Bacteria that are challenging to combat have drawn a lot of attention in recent century as society has developed. Examples include infections in the biomedical field, contamination of drinking water, and biological invasion in the environmental engineering field, all of which pose a hazard to the survival of all kinds of animals in nature and human health [1,2,3,4,5]. Traditional disinfection methods, including chlorine (Cl), ultraviolet light (UV), and ozone, can eliminate microorganisms in wastewater, and researchers also have developed new sterilization methods, such as photocatalysis, new materials, and combined disinfection processes, which have shown excellent results [2,6,7]. Meanwhile, in biomedicine, the use of antibiotics has played an important role in addressing bacterial infections in humans [8].

Nevertheless, there are limits to these approaches. For example, low antibacterial efficiency, high cost, drug resistance, and secondary pollution in water [9,10,11]. Most accepted methods used to fight microorganisms do not effectively remove stubborn microorganisms to meet the current requirements regarding water quality and the health of the human body, and better methods are needed [2,3,12,13,14]. Nowadays, many researchers have proposed reactive oxygen species (ROS) antimicrobial methods for biomedical and environmental engineering applications. In recent years, a new concept of photodynamic therapy (PDT), which can produce cytotoxic ROS to attack microorganisms in the presence of photosensitizers (PSs) and oxygen by an appropriate excitation light, has been gaining popularity in the medical field [15]. Qi et al. reviewed the antimicrobial photodynamic therapy of nanostructured materials encasing PSs against oral bacterial biofilms and infectious diseases [16]. Chemodynamic therapy (CDT) is also a new antimicrobial strategy, which can produce •OH through Fenton or Fenton-like reactions to kill microorganisms. Zhang et al. prepared ZnO_2_-Cu @RB NPs using copper-doped zinc peroxide nanoparticles (ZnO_2_-Cu NPs) and the antibacterial agent Rose Bengal (RB), efficiently killing *S. mutans* in acidic biofilms by CDT [17]. Among numerous ROS, •OH is widely used. At the same time, in environmental engineering, advanced oxidation processes (AOPs) were developed to extend the application of •OH, which refer to emerging and widely applicable water purification technologies [18,19,20,21]. For example, Zaidan et al. showed that the heterogeneous catalyst TiO_2_ reduced the toxicity of phenol and its intermediates through advanced oxidation processes (AOPs) while exhibiting antimicrobial effects [22]. Liu et al. reviewed the mechanism, advantages, and disadvantages of AOPs and their combination with nano-catalyzer in the treatment of textile dye wastewater and pharmaceutical residue wastewater [23]. Napoleao et al. demonstrated through a series of experiments that AOPs could degrade 71.9% to 100% of the pollutants in sewage [24]. Meanwhile, it was hoped that AOPs could remove stubborn microorganisms and would impact the removal of microorganisms mainly through •OH, a strong and non-selective chemical oxidizer [6,25,26]. •OH attacks microorganisms, which has a high reaction speed and can avoid secondary pollution, showing an excellent degradation performance [27].

However, the short half-life of •OH is an issue [28]. Compared to •OH, sulfate radicals, which are easily ignored among radicals, have shown an excellent performance. Xiao et al. summarized the advantages of SO_4_^−^· over •OH: (1) SO_4_^−^· has higher oxidation than •OH, and its oxidation potential (2.5–3.1 V) exceeds •OH (1.8–2.7 V). (2) SO_4_^−^· has a longer lifetime (30–40 µs for SO_4_^−^· and 1 µs for •OH). (3) SO_4_^−^· has wider application conditions and a higher degradation efficiency [28]. Recently, based on AOP, sulfate radical-based AOPs (SR-AOPs) have emerged [29,30]. Some researchers believe that SO_4_^−^· kills microorganisms by destroying their cell membranes, walls, and genetic material [31]. The application of SR-AOPs in sterilization and disinfection has received a lot of attention and is being actively researched due to its extraordinary properties [32,33,34,35,36,37,38,39,40]. For instance, Rodríguez-Chueca et al. applied cobalt ferrite nanoparticles as a nano-catalyst to activate peroxymonosulfate, creating SO_4_^−^·. Under UV radiation, the number of different bacteria, such as *Escherichia coli (E. coli)* and *Enterococcus*, could be reduced and even completely inactivated within 30–60 min in the simulated wastewater samples [41]. Zhang et al. used sulfide micron zero-valent iron to activate persulfate to produce SO_4_^−^·, showing a shocking removal effect on resistant *E. coli* and drug resistance genes (ARG) [42]. Xiao et al. used zero-valent iron (ZVI) to activate peroxydisulfate (PDS) to generate SO_4_^−^· and studied SO_4_^−^·-mediated Gram-negative (*E. coli*) and Gram-positive (*enterococcus faecalis*) inactivation, aiming to elucidate the profile and mechanism of sulfate radical-mediated disinfection of microorganisms in sewage [43].

Typically, SO_4_^−^· can be produced by activating two of the strongest precursor peroxides: peroxymonosulfate (PMS) and peroxydisulfate (PDS), which is shown in Equations (1) and (2) [28]. Common activation methods include metal or non-metal catalysts, photo-catalytic activation (h^+^/e^−^), UV, heat, ultrasound, microwave, and alkali [32,44,45,46,47,48]. For example, the transition metal can change from a low to a high state to produce sulfate radicals by breaking the O-O bond of the persulfate (Equations (3) and (4)) [28,32]. At present, the most reliable way to activate PDS is the use of a catalyst because microwave and ultrasound are difficult to use in the treatment of water environment with a large area; the activation efficiency of light, heat, and alkali is low; and the environmental benefits are poor [28]:

S_2_O_8_^2−^ → 2 SO_4_^−^· (1)

HSO_5_^−^ →SO_4_^−^· (2)

M^n+^ + S_2_O_8_^2−^ →SO_4_^−^· + M^(n+1)+^ + SO_4_^2−^ (3)

M^n+^ + HSO_5_^−^ →SO_4_^−^· + M^(n+1)+^ + OH^−^ (4)

In recent years, many studies have applied nano-catalysts to the activation of PDS/PMS, exhibiting a variety of advantages, principally ascribed to their specific nanostructure and the larger specific area, in addition to high selectivity, high recoveries, and widespread optical properties. Another important advantage is the adjustability of the catalytic activity by changing its morphology, such as the size and shape, and applying external stimuli such as light and sound waves [49]. These nanomaterial design methods involving SO_4_^−^· are promising techniques for the removal of microorganisms from wastewater and treatment of bacterial infections and are deemed prospective alternative approaches for environmental remediation and biomedicine [50]. For instance, Huang et al., Han et al., Xiong et al., Tian et al., and Zhao et al. reviewed Mn-based catalysts, metal–organic frameworks (MOFs), and biochar-based catalysts, respectively, which could activate PMS/PDS to produce SO_4_^−^· [51,52,53,54,55]. In addition, Giannakis et al. and Ushani et al. discussed the application of SR-AOPs in various wastewater and environmental remediation scenarios [32,56]. Moreover, Kurian et al. also summarized the use and progress of nanomaterials in AOPs [57]. However, at present, few researchers have systematically summarized the various types of nanomaterials that can activate PMS/PDS to produce SO_4_^−^· and their applications in antimicrobial fields. Furthermore, there is a lack of research on disinfection in the field of environmental remediation and anti-infection and antitumor properties in the field of biomedicine.

**Figure 1:**
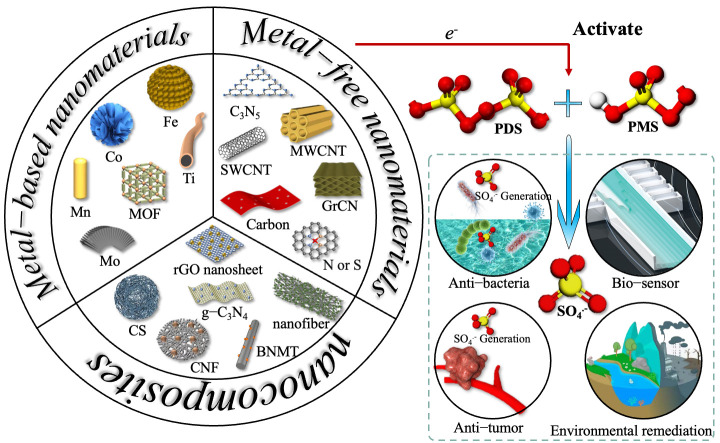
Three categories of nanomaterials used to activate PDS/PMS to generate SO_4_^−^· and their applications in environmental remediation and biomedicine. Reprinted with permission from [58], Copyright 2021, American Chemical Society.

Therefore, in this article, the latest designs of nanomaterials that can effectively activate PMS/PDS to produce SO_4_^−^· are reviewed, which are divided into three categories: metal-based nanomaterials, metal-free nanomaterials, and nanocomposites. In addition, we discuss the potential mechanisms by which these nanomaterials activate PMS/PDS and the applications of SO_4_^−^· in the environmental remediation and biomedical fields (Figure 1). Finally, further research opportunities for the practical application of nanomaterials are identified and discussed.

## 2. Metal-Based Nanomaterials

Metal-based nanomaterials provide electrons for PMS/PDS to be activated to generate SO_4_^−^·, mainly through the change in the valence state, which has shown a high activation efficiency [28]. The metallic elements include Co, Fe, Mn, Ni, Mo, Ti, V, Ru, Cu, etc. According to many researchers, the morphology of metal-based nanomaterials is easy to change and exhibits excellent Fenton-like behavior. Additionally, magnetic nanoparticles can be easily separated from water, which prevents the occurrence of secondary water pollution in the environment. However, metal leaching can still lead to inactivation of the activator, and agglomeration may reduce the degradation efficiency.

**Figure 2:**
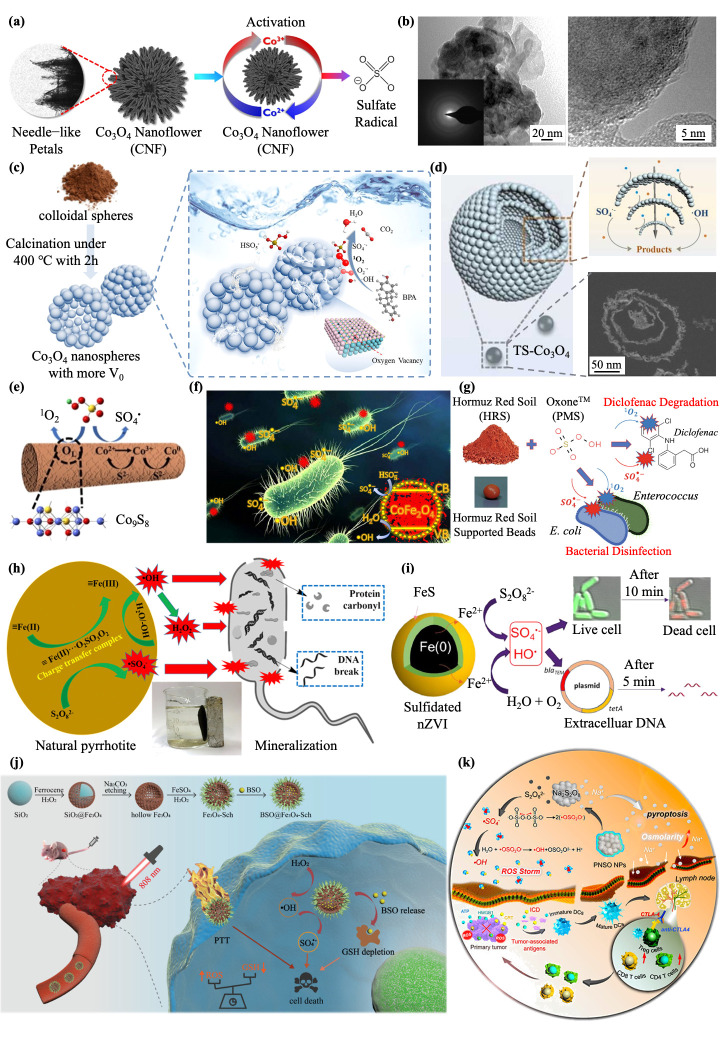
(**a**) Enhanced degradation of paracetamol in water using sulfate radical-based advanced oxidation processes catalyzed by three-dimensional Co_3_O_4_ nanoflower. Reprinted with permission from [60], Copyright 2019, Elsevier. (**b**) TEM of the amorphous structure CoO-A. Reprinted with permission from [61], Copyright 2021, Elsevier. (**c**) Schematic illustration of oxygen vacancies introduced into the Co_3_O_4_ nanoparticles. Reprinted with permission from [62], Copyright 2020, Elsevier. (**d**) Schematic illustration of hollow multi-shelled Co_3_O_4_. Reprinted with permission from [63], Copyright 2022, Elsevier. (**e**) Schematic illustration of S doping in Co_3_O_4_ to realize the catalytic cycle between Co^3+^/Co^2+^/Co^0^. Reprinted with permission from [64], Copyright 2021, Elsevier. (**f**) Schematic illustration of the use of magnetic spinel CoFe_2_O_4_ nanoparticles to activate PMS in the inactivation studies of two pathogenic bacteria, *E. coli* and *Enterococcus*. Reprinted with permission from [41], Copyright 2020, Elsevier. (**g**) Schematic illustration of the use of HRS as a nano−catalyst to activate PMS to produce SO_4_^−^· and kill *E. coli* and *Enterococcus.* Reprinted with permission from [66], Copyright 2021, Elsevier. (**h**) Schematic illustration of the use of natural magnetic pyrrhotite (NP) as a catalyst to activate PDS to kill microbials. Reprinted with permission from [67], Copyright 2017, Elsevier. (**i**) Schematic illustration of the synergistic effect of sulfidated nano zerovalent iron and persulfate on the inactivation of antibiotic-resistant bacteria and antibiotic resistance genes. Reprinted with permission from [69], Copyright 2021, Elsevier. (**j**) GSH-depleted nanozymes with dual-radical enzyme activities for tumor synergic therapy. Reprinted with permission from [76], Copyright 2021, Wiley. (**k**) Schematic illustration of the use of Na_2_S_2_O_8_ nanoparticles as a novel ROS generator, which treats tumor by gradually generating SO_4_^−^·. Reprinted with permission from [77], Copyright 2020, American Chemical Society.

Co-based nano-catalysts can activate PMS/PDS to produce sulfate radicals by switching between Co^2+^ and Co^3+^, which can fight bacteria and purify sewage [41,59]. The performance of cobalt-based nano-catalysts in activating PMS/PDS is also highly dependent on the structure and morphology. Different morphology of cobalt nanomaterials has been reported, for instance, nanotubes, nano-rings, nanospheres, nanosheets, and nanoplates [28]. However, cobalt oxide nanoparticles often aggregate and the catalytic activity of nanoparticles with this morphology is also unsatisfactory. In recent years, researchers have enhanced the catalytic activity in different ways. Yun et al. synthesized Co_3_O_4_ nanoflower structures to increase the active sites by increasing the surface area and reducing aggregation, showing better catalytic activity, and achieving better degradation of paracetamol in wastewater (Figure 2a) [60]. In addition, in comparison to the common Co-based crystal structure, the newly designed CoO-A has an excellent PMS activation efficiency in the removal of the environmental pollutant parachlorophenol (4-CP) because of its amorphous structure (Figure 2b) [61]. To address the lack of oxygen vacancies due to the bulk aggregation of the materials, oxygen vacancies are introduced into Co_3_O_4_ nanoparticles by thermal treatment to promote charge transfer capability and electrical conductivity, which is also used to increase the PMS activation capacity for the degradation of recalcitrant organic pollutants (bisphenol A, BPA) (Figure 2c) [62]. Ma et al. designed a hollow three-shell Co_3_O_4_ structure to lessen the reunions of the nano-catalyst and hold the catalytic site, thus balancing the contradiction between the stability and reactivity of the nanoparticles, which is crucial for the advanced oxidation processes (AOPs) of PMS activation in wastewater treatments (Figure 2d) [63]. Moreover, the introduction of non-metal heteroatoms could be an effective means to enhance the catalytic performance of nano-catalysts to PMS. Zhou et al. doped S into Co_3_O_4_, realizing the catalytic cycle between Co^3+^/Co^2+^/Co^0^, and allowing an increase in the PMS reaction with Co^2+^ to generate SO_4_^−^· (Figure 2e) [64]. In order to improve the cycle of Co^3+^/Co^2+^ and achieve bimetallic synergism, Rodríguez-Chueca et al. designed magnetic spinel CoFe_2_O_4_ nanoparticles to activate PMS for inactivation studies of two pathogenic bacteria: *E. coli* and *Enterococcus*. The results showed that the coexistence of 0.2 mM PMS and 0.05 mg/L CoFe_2_O_4_ could reduce the bacteria in the wastewater and achieve complete inactivation in 30–60 min under UV radiation (Figure 2f) applied to the disinfection of wastewater [41]. In order to increase the stability of cobalt, Osias et al. used β-zeolite to support cobalt ions as a catalyst for the activation of PMS to produce SO_4_^−^·, showing an excellent catalytic performance and a more obvious degradation effect of methylene blue in wastewater [65].

Many studies about the use of Fe-based nanomaterials for the activation of PMS/PDS have been carried out due to their favorable activity, reduced contamination, and lower cost [28]. Soil is rich in natural iron (hydrogen) oxides and minerals, such as goethite ilmenite (FeTiO_3_), mackinawite (FeS), pyrite (FeS_2_), magnetite hematite (γ-Fe_2_O_3_), Hormuz Red Soil (HRS) (α-Fe_2_O_3_), and FeO (OH). Some have been shown to be native catalysts for the activation of PMS/PDS to remove contaminants and kill bacteria. Mohammadi et al. used HRS as a nanocatalyst to activate PMS to produce SO_4_^−^·, killing *E. coli* and *Enterococcus* in a relatively short time, and showing potential for wastewater purification and disinfection (Figure 2g) [66]. Xia et al. used natural magnetic pyrrhotite (NP) as a catalyst to activate PDS to kill microbial contaminants in water. It has also been proposed that the mechanism of bacterial death is through increased envelope lesions that exacerbate intracellular protein depletion and genomic damage. In addition, the NP/PDS system also showed good inactivation efficiency for *E. coli* in real water substrates such as surface water and secondary sewage, providing a good idea for the disinfection of wastewater (Figure 2h) [67]. In recent years, nano-zero-valent iron (NZVI) has been widely researched to achieve effective water pollution remediation due to its high reactivity, resulting from its high reduction [42]. Nevertheless, NZVI nanoparticles tend to aggregate and are easily corroded, resulting in a rapid loss of reactivity. Interest in the vulcanization of NZVI with reduced sulfur compounds in order to maintain reactivity and longevity has increased [68]. Yu et al. designed a core-shell structure using FeS as a shell, which enabled S-NZVI to facilitate electron transfer for better activation of PDS. The complete inactivation of antibiotic-resistant bacteria (ARB) and the efficient removal of antibiotic resistance genes (ARGs) by S-NZVI/PDS were also demonstrated in real drinking water and real wastewater effluent that contained natural organic matter and suspended solids (Figure 2i) [69]. At the same time, the ability of naked and three modified NZVI, namely sulfide-modified NZVI, Ni/NZVI, and activated carbon-loaded NZVI, to remove contaminants in PMS activation was compared, and Ni/NZVI stood out. Here, the introduction of Ni alleviated surface passivation, enabled the supply of active radicals through the electric couple effect between NZVI and Ni^0^, and enhanced the Fe^2+^/Fe^3+^ transition for improved removal of sulfamethazine in aqueous solution [70]. Surprisingly, studies have found that Fe (IV) can be produced in the NZVI/persulfate system, which is more selective, has a longer service life, and provides greater contact with underground target pollutants than highly reactive radicals (SO_4_^−^· and •OH). However, the value of Fe (IV) deserves further exploration [71]. It has also been proposed that a moderate amount of Fe^2+^ can activate bisulfite to generate SO_4_^−^· while an excessive amount of Fe^2+^ can quench SO_4_^−^·. NZVI is a more potent and durable bisulfite activator by gradually generating Fe^2+^, avoiding excess Fe^2+^ quenching SO_4_^−^·, such as Fe^2+^ + SO_4_^−^· → SO_4_^2−^ + Fe^3+^, revealing that the NZVI/bisulfite system has significant potential for triphenyl phosphate (TPHP) elimination in waterbodies [72]. In addition to the Fe-based nanomaterials mentioned above, Fe_3_O_4_ is also a widely studied nanomaterial that cannot be ignored. However, Fe_3_O_4_ tends to aggregate due to the strong magnetic attraction between the nanoparticles. Therefore, Zhan et al. [73] and Cai et al. [74] achieved a better catalytic performance using β-cyclodextrin (β-CD), ethylenediamine tetraacetic acid (EDTA), or humic acid (HA) as enhancers to obtain a better activation performance, providing novel materials for use in the Fenton-like process for the degradation of contaminants. Analogously, Tan et al. designed EGCG-modified Fe_3_O_4_ to effectively promote PMS activation due to the alleviation of agglomeration after the EGCG modification, providing new insights for the decomposition of organic pollutants dissolved in water [75]. Other researchers have also constructed an Fe_3_O_4_–Schwertmannite nanocomposite (Fe_3_O_4_-Sch). Surprisingly, SO_4_^2−^ is converted to SO_4_^−^· when subjected to a massive •OH attack, serving as a high-performance SO_4_^−^· producer. In particular, this is one of the rare cases in which PDS cannot be employed as a precursor to generate SO_4_^−^·, for which dual-free radical combination therapy showed an overwhelming tumor suppressive effect, resulting in infinite possibilities for the application of SO_4_^−^· in tumor treatment (Figure 2j) [76]. Researchers have synthesized Na_2_S_2_O_8_ nanoparticles as novel ROS generators, which treat tumors by gradually generating SO_4_^−^· and •OH in situ through Na_2_S_2_O_8_, which is undoubtedly significant progress that cannot be ignored in the application of SO_4_^−^· in tumor treatment (Figure 2k) [77].

**Figure 3:**
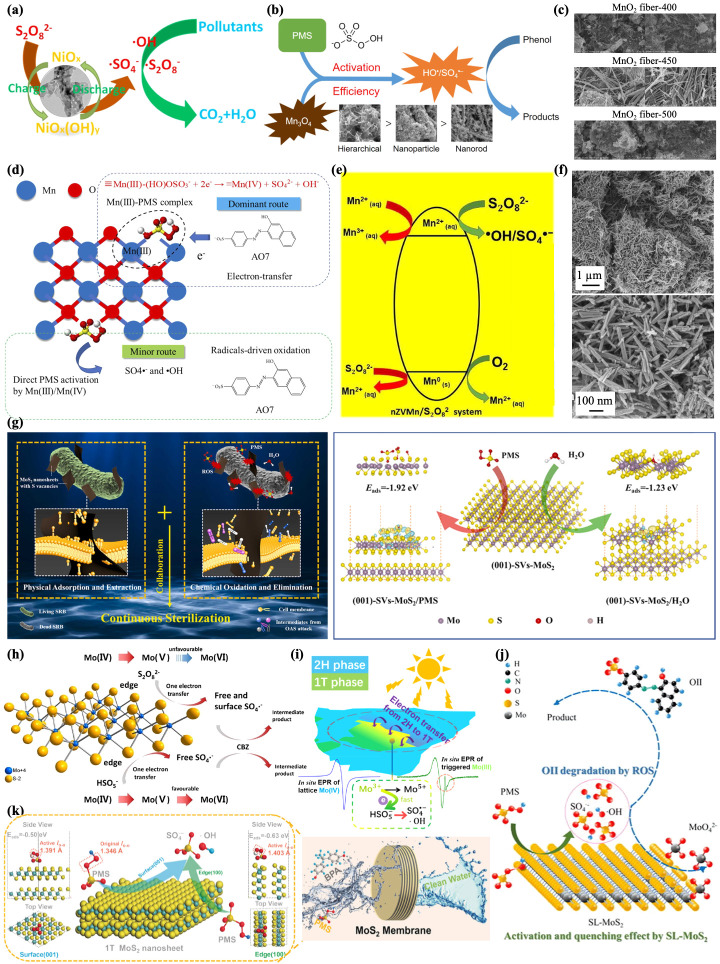
(**a**) Schematic illustration of the use of NiO nanosheets rich in porous defects for PDS activation. Reprinted with permission from [78], Copyright 2019, Elsevier. (**b**) Three-dimensional graded Mn_3_O_4_-H nanomaterials assembled from 2D nanosheets with a 10–20 nm thickness to activate PMS. Reprinted with permission from [79], Copyright 2019, Elsevier. (**c**) TEM of different sized and shaped MnO_2_ nanofibers and nanowires. Reprinted with permission from [80], Copyright 2021, Elsevier. (**d**) Schematic illustration of amorphous MnOx. Reprinted with permission from [81], Copyright 2022, Elsevier. (**e**) Schematic illustration of nano-zerovalent manganese (nZVMn, Mn^0^) gradually providing Mn^2+^ for efficient utilization of PDS. Reprinted from. Reprinted with permission from [82], Copyright 2019, Elsevier. (**f**) TEM of a nanoscale manganese oxide octahedral molecular sieve (OMS-2) with the structure of cryptomelane. Reprinted with permission from [83], Copyright 2019, Springer Nature. (**g**) Schematic illustration of nanokillers’ activation by PMS showing efficient anaerobic microorganism disinfection. Reprinted with permission from [86], Copyright 2022, Elsevier. (**h**) Schematic illustration of Mo (IV) in MoS_2_ nanosheets converted into high-price Mo (V) and Mo (VI). Reprinted with permission from [84], Copyright 2020, Elsevier. (**i**) Schematic illustration of MoS_2_ 2H/1T heterojunctions. Reprinted with permission from [92], Copyright 2019, American Chemical Society. (**j**) Schematic illustration of neatly arranged MoS_2_ nanosheet stacking structures. Reprinted with permission from [93], Copyright 2022, Elsevier. (**k**) Schematic illustration of a layered film made of a stacked two-dimensional structure. Reprinted with permission from [94], Copyright 2019, Wiley.

Because the redox potential of Ni/Ni^2+^ is not as good as that of Co^2+^/Co^3+^ or Fe^0^/Fe^3+^ in activating PMS/PDS, Ni^2+^/Ni-based catalysts for the generation of SO_4_^−^· have rarely been studied. Encouragingly, however, Yue et al. synthesized NiO nanosheets rich in porous defects for PDS activation, providing a new Ni-based catalyst for persulfate activation, which represents a promising Fenton-like catalyst for environmental remediation (Figure 3a) [78].

Mn-based nanomaterials have also been considered meritorious catalysts in activating PMS/PDS, and in contrast to cobalt, manganese oxides (MnOx) are abundant in nature and are more compatible with the environment [28]. Nanorods, nanotubes, nanowires, α-MnO_2_ (such as sea urchin), and δ-MnO_2_ (such as corolla) have previously been investigated, whose ability to activate PMS depends on their morphology, valent state, and crystalline phase. Compared to Mn_3_O_4_ nanoparticles or nanorods, Wang et al. developed 3D-graded Mn_3_O_4_-H nanomaterials assembled from 2D nanosheets with a 10–20 nm thickness, revealing a higher catalytic performance in the activation of PMS to remove organic pollutant phenol in wastewater, which was attributed to the larger surface area and increased number of active sites generated (Figure 3b) [79]. At the same time, Bi et al. changed the size and shape of MnO_2_ nanomaterials to form MnO_2_ nanofibers and nanowires with an enhanced surface area and surface oxygen vacancies to better control the activation of PMS by adjusting the reaction temperature and time during acid treatment. This study demonstrated improved catalytic activity and recyclability for the oxidative degradation of various organic dyes such as tetracyclines and bisphenol A and showed the potential for water remediation (Figure 3c) [80]. In addition to the well-crystallized-phase MnOx, Zhu et al. investigated the rarely reported amorphous MnOx (AMO), which has exhibited potent PMS activation properties and has potential applications in wastewater treatment (Figure 3d) [81]. In addition, Similar to NZVI, Shah et al. investigated nano-zerovalent manganese (nZVMn, Mn^0^), which can gradually provide Mn^2+^ for the efficient utilization of PDS, showing an outstanding activation efficiency. Additionally, it was found to be stable and environmentally friendly in the treatment of CIP, which is a promising alternative for potential remediation and detoxification of CIP and other emerging water pollutants (Figure 3e) [82]. In addition, Wei et al. proposed that the presence of different valence states of Mn could exhibit a better catalytic performance. They designed a nanoscale manganese oxide octahedral molecular sieve (OMS-2) with the structure of cryptomelane (Figure 3f), and manganese (+2, +3, +4) with different valence states coexisted on the side of the octahedron when activating PMS (Equations (5) and (6)). The fine degradation efficiency of phenol in different water sources and the stability after continuous use indicated the possible application of PMS/OMS-2 in real wastewater treatment [83]:

Mn^3+^ + HSO_5_^−^ → Mn^4+^ + SO_4_^−^· + OH^−^ (5)

Mn^4+^ + HSO_5_^−^ → Mn^3+^ + SO_5_^−^ + H^+^ (6)

MoS_2_ is a layered transition metal disulfide that is multiphase and multivalence, which has excellent PDS/PMS catalytic activity due to its high activity and superior two-dimensional structure [84]. For example, Chen et al. studied two-dimensional nanosheet MoS_2_, in which the sulfur vacancies activate PMS to produce SO_4_^−^, which can extract lipids from cell membranes and subsequently destroy them. This showed almost a 100% *E. coli* sterilization rate within 5 min, representing an innovative “poison arrowhead” approach for disinfection [85]. At the same time, Wang et al. proposed a similar idea and verified that it could achieve rapid and efficient sterilization in various environments, which provides new possibilities for fighting microbes in the environment (Figure 3g) [86]. In addition, Mo (IV) in MoS_2_ nanosheets was converted into high-price Mo (V) and then reoxidized to Mo (VI), which changed PMS to form SO_4_^−^·, where S was involved in formatting the reactive layered structures that enhanced the activity. However, the process of reducing Mo (VI) to a lower valence may be more difficult than that of oxidation, and it is unfavorable for the MoS_2_/PMS/PDS system (Figure 3h) [84]. Heteroatom doping of MoS_2_ is an effective way to improve its activation ability by depressing its high potential. Regarding non-metallic atomic (B, P, N) doping, tuning of the electronic properties helps to generate additional active unsaturated sulfur sites [87,88]. Compared to non-metallic atom doping, MoS_2_ can produce new bimetallic active sites with sulfur bridges due to Co, Fe, and Ni atomic doping (especially Co doping), that is, the catalytic performance is greatly improved [89,90]. Zeng et al. designed defect-rich Co-N double-doped MoS_2_ anchored in biomass carbon, which showed an astounding synergistic heteroatom effect to expose many active sites, allowing accelerated electronic transfer, enhancing the activation ability, and accelerating the mineralization and degradation of the target environment pollutant. It is striking that the excellent activation capability of self-born in situ SO_4_^−^· (S (VI) activated to SO_4_^−^) was also demonstrated [91]. At the same time, MoS_2_ has two major crystal phases: 1T and 2H. Due to their different orbital occupancies, the 1T phase is the metal phase while the 2H phase is semiconducting. Chen et al. constructed 2H/1T heterojunctions, which allowed light absorption and electronic production in 2H and facilitated the activation reaction of 1T-MoS_2_ edge sites through the effective charge separation at the interface through visible light (Figure 3i) [92]. In addition, it has been proposed that MoS_2_ nanosheets have a dual role, namely the activation of PMS and quenching of radicals. In order to weaken the ROS quenching effect, Li et al. designed neatly arranged MoS_2_ nanosheet stacking structures to protect the oxidized surface/edge to achieve better chemical stability and adsorbability, and thus showed an excellent orange II (OII) degradation performance (Figure 3j) [93]. In order for short-lived active free radicals to exert oxidation properties effectively, MoS_2_ could also be designed as a layered film made of a stacked two-dimensional structure with adjustable spacing as a catalyst. The nonlinear transmission between MoS_2_ nanolayers leads to water having high diffusivity so that the short-lived reactive radicals efficiently oxidize contaminants to treat persistent environment pollutants (Figure 3k) [94]. Moreover, some researchers have designed MoS_2_/PDS loaded onto a carbon cloth as a flexible photothermal film that rapidly converts near-infrared light into heat, which activates PDS, demonstrating the potential of applying light-to-heat conversion in Fenton-like processes for pollution control. This opens new avenues towards the utilization of inexhaustible solar energy and novel approaches for environmental remediation [95]. Shockingly, Wang et al. prepared a sensitive terylene system of polyethyleniminized perylene derivatives ptc-pei, S_2_O_8_^2−^, and 3D floral MoS_2_ (3D MoS_2_ NFs), based on the fact that MTX of methotrexate could be converted to other substances by SO_4_^−^·, leading to quenched ECL signals. The prepared sensor could achieve highly sensitive and accurate MTX determination with excellent results [96]. At the same time, ECL signaling was enhanced through SO_4_^−^·, which acted as a folic acid (FA) sensor [97] and was applied for insulin detection [98], showing its potential for biosensing medicine.

**Figure 4:**
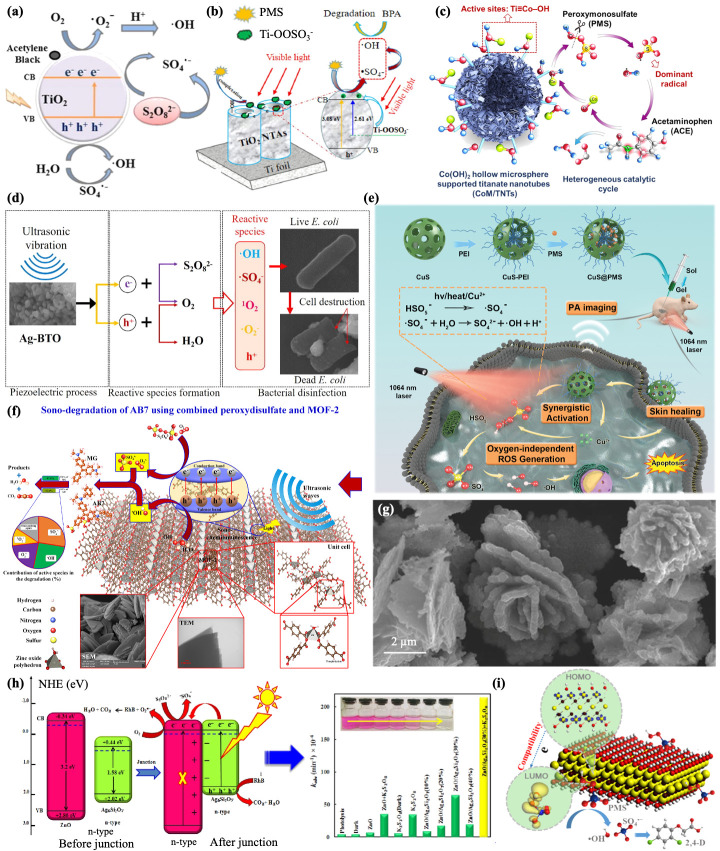
(**a**) Schematic illustration of the bandgap of TiO_2_ doping with acetylene black. Reprinted with permission from [101], Copyright 2020, Elsevier. (**b**) Schematic illustration of the use of the visible light (VL)-responsive nanotube array (TiO_2_NTAs) for PMS activation. Reprinted with permission from [103], Copyright 2020, Elsevier. (**c**) Schematic illustration of titanate nanotubes (TNTs) for PMS activation. Reprinted with permission from [105], Copyright 2020, Elsevier. (**d**) Schematic illustration of the use of silver−doped barium titanate (Ag-BTO) as a catalyst for the activation of PDS to produce SO_4_^−^· and destroy the cell membrane of *E. coli*. Reprinted with permission from [106], Copyright 2020, Elsevier. (**e**) Schematic illustration of the use of CuS @PMS NPs to generate SO_4_^−^· to form a stimulus in response to oxygen-independent tumor nanotherapy. Reprinted with permission from [111]. (**f**) Schematic illustration of the use of MOF-2 as a semiconductor for PDS activation. Reprinted with permission from [114], Copyright 2020, Elsevier. (**g**) TEM and mapping of nanomaterials containing three metallic elements (Cu, Fe, Sn). Reprinted with permission from [121], Copyright 2019, Elsevier. (**h**) Schematic illustration of ZnO/Ag_6_Si_2_O_7_ heterojunctions’ construction. Reprinted with permission from [141], Copyright 2020, Elsevier. (**i**) Schematic illustration of the use of Ti_3_C_2_T_x_ (MXene) for PMS activation. Reprinted with permission from [153], Copyright 2020, American Chemical Society.

TiO_2_ is a cost-effective, stable, and environmental photocatalyst that has received significant attention in the field of water environment restoration. It simultaneously produces oxidized holes (h VB^+^) at the valence band and reducing electrons (e CB^−^) at the conduction band when activated by light. However, the rapid recombination of both notably depresses the oxidation capacity of the photocatalyst. The addition of PDS can restrain the recombination of h VB^+^ and e CB^−^ while producing SO_4_^−^· [99,100]. Doping with metallic or nonmetallic materials reduces the band gap to distinctly improve the photocatalytic activity of the greater light absorption regions. Inorganic oxidants can play the role of electron traps to prevent the recombination of h VB^+^ and e CB^−^. An improvement in the adsorptivity and PDS productiveness of TiO_2_ via doping with acetylene black has been demonstrated, thus effectively diminishing the aquatic toxicity of tetracycline hydrochloride (TH) (Figure 4a) [101]. This could also be achieved by incorporating the organic molecule diimide derivative 2,5-double (tributyl methionyl) thiophene-diimide-T (Bis-PDI-T) into titanium dioxide to form a heterojunction, expanding the range of the optical response, and improving the separation and transmission productivity of photogenerated electron [102]. TiO_2_ nanotubes also have bright spots, and the visible light (VL)-responsive nanotube array (TiO_2_NTAs) could effectively activate PMS. The TiO_2_ NTAs/PMS/VL system displayed enhanced BPA degradation in tap water and drinking water compared with deionized water, providing new insight into PMS activation in practical water treatment (Figure 4b) [103]. Well-aligned and dense and homogeneous arrays of blue-TNA (blue-TiO_2_ nanotubes) were used as anodes to form Ti^3+^, which directly generated SO_4_^−^· via the reaction at a high anodic potential in Na_2_SO_4_ solution: SO_4_^2−^ → SO_4_^−^· + e^−^. Ti^3+^ generated a series vacancy belt of the electronic state, which resulted in the highly efficient degradation of the organic pollutants, indicating that it is a key opportunity for the treatment of organic wastewater [104]. Recently, titanate nanotubes (TNTs) have been studied for the decoration of framework activation nanomaterials. The stable active site was due to the hollow nanotubular structure and sizeable specific area (up to 240.20 m^2^/g). Moreover, as a cationic adsorbing material, it was able to easily adsorb the transition element in the intercalation layer. Additionally, the massive •OH in TNT promoted the formation of Co (OH)^+^, which is a fundamental substance for PMS activation (Figure 4c) [105]. Xia et al. studied the formation of electron hole pairs by ultrasonic piezoelectric catalysis using silver-doped barium titanate (Ag-BTO) as a catalyst, which could effectively activate PDS to produce SO_4_^−^·, and then destroy the cell membrane of *E. coli*. Ultrasound also accelerated the penetration of SO_4_^−^·, enhancing the subsequent internal metabolic dysfunction and enzymatic oxidation of SO_4_^−^·, which provides new ideas for combating microorganisms in living organisms and the environment (Figure 4d) [106].

The performance of V_2_O_5_ activation of PDS to degrade organic pollutants is strongly influenced by the morphology, with some studies concluding that VW (linear) > vs. (spherical) > VR (rod). Furthermore, the surface area and exposed crystalline surfaces may affect the activation capacity and a lower Fermi energy and higher VW positive charge density favors activation [107]. RuO_2_ (with chemical stabilization and high electroconductivity) could act as a PMS activator. Two-dimensional RuO_2_ nanosheets are superior to nanoparticles and nanorods in terms of the electrical conductivity, active sites, superficial area, and endurance, achieving maximum catalytic performance. Although costly, they are superior to transition metal oxides. For instance, they show chemical stability in a wide pH range, low leaching, high PMS activation efficiency, universal handling of various organic substances, high-speed electron transfer on nanosheets, and good interacting friendly surfaces with PMS. By attempting to reuse it as many times as possible, this could compensate for its high costs [108]. Tungstate nanomaterials with an HTB structure could strike W^6+^ and restore it to W^5+^ to store light-born electrons under light excitation, and the reduced rubidium tungstate Rb_4_W_11_O_35_ (rRT)-containing nanomaterials of NaBH_4_ significantly aggrandized the active sites, thus enhancing the electroconductivity and electricity conduction. Rubidium tungstate (RRT) nanorods have been shown to activate PDS under alkaline conditions to effectively remove bisphenol A (BPA), providing new insights into the development of alkaline persulfate-activation nanocatalysts for industrial wastewater treatment [109]. Since copper ions have diversified valence states, such as Cu^3+^, Cu^2+^, Cu^+^, and Cu^0^, copper nanorods and some irregular nanoparticles can activate PDS to produce SO_4_^−^· for the decolorization of diazo dye in wastewater, assisting in predicting their fate in the environment [110]. In addition, CuS @PMS could be formed by loading PMS in hollow mesoporous CuS NPs to generate SO_4_^−^·, forming a stimulus in response to oxygen-independent tumor nanotherapy. In a previous report, the ubiquitous hypoxia and insufficient hydrogen peroxide (hydrogen peroxide) content in the tumor microenvironment inevitably hindered effective production of ROS. However, this novel nanocomposite could show a significant tumor suppressive effect by producing SO_4_^−^· in an anaerobic microenvironment, which provides a solid foundation for its later application in biological tumor treatment (Figure 4e) [111]. Oh et al. prepared three-dimensional layered CuBi_2_O_4_ composites so that the synergic coupling effect between CuBi_2_O_4_ and the deposited metal oxides helped to activate the generation of SO_4_^−^· in PMS for *E. coli* disinfection [112]. To make the Cu-based catalyst more dispersed and stable, Tang et al. prepared Cu_2_O @NC by anchoring Cu_2_O to a nonmetallic carbon nanocatalyst, which exhibited activation of PMS. It had strong removal properties for various drugs, such as tetracycline (TC) and amoxicillin (AMX), providing a fast, efficient, and feasible way for treating operating room sewage and reducing the possibility of bacterial resistance [113].

Metal–organic frameworks (MOFs) are hybrid materials formed by organic and inorganic units connected by strong bonds, making use of the advantages of both organic and inorganic compounds. In recent years, MOFs have attracted great interest from researchers due to their excellent properties, including a large specific surface area and suitable pore structure, in addition to the crystalline form of MOFs, which have powerful chemical bonds, specific geometries, and adjustable connecting units. For example, MOF-2 as a semiconductor generates e^−^/h^+^ pairs under ultrasonic excitation, which could activate PDS (Figure 4f) [114]. Perylene-34,910-tetracarboxylic diimide (PDINH), a semiconductor organic photocatalyst with a suitable band gap and high electron mobility and affinity, compounded with MIL-88A(Fe) could improve the PDS activation efficiency, exhibiting an outstanding degradation performance toward chloroquine phosphate (CQ), which offers deep insights into the mechanisms of organic pollutant degradation via photocatalysis-activated SR-AOPs over Fe-MOF photocatalyst [115]. The zeolite imidazolate framework (ZIF) is thermally and chemically stable, and zinc-based metal–organic framework (ZIF-8) nanostructures have been shown to act as activators for PDS under sonication [116].

In recent years, researchers have combined two or three metallic elements with excellent properties mainly from the synergy of different elements (Figure 4g), increase in active sites, enrichment of oxygen vacancies, optimization of nanostructures, extension of the lifetime by promoting cycling between M^n^ and M^n+1^, improvement in stability by reducing agglomeration and reducing the leaching of metallic elements, and improvement of the recoverability by increasing magnetic properties, etc., which are summarized in Table 1. This includes a spinel ferrite MFe_2_O_4_, with exceptional superparamagnetism; a large adsorption capacity; and biological, optical, and catalytic properties [117]. Additionally, metal-doped MOFs are included, which have been thoroughly investigated for their effective catalytic activity.

Recently, the construction of heterojunctions to promote hole-electron separation and inhibit complexation of the carrier to improve the catalytic performance has also been widely studied, such as ZnO/Ag_6_Si_2_O_7_ (Figure 4h) [141], etc., as shown in Table 2. 

Similar to the heterojunction principle, the visible light capture capability of ZnO is insufficient due to the wide band gap energy, and in addition, the quantum efficiency of ZnO suffers from the strong effects of rapid electron-hole recombination. A good strategy is the use of ferrite silver (AgFeO_2_) with a narrow energy gap to improve the photocatalytic capability of ZnO [150]. Similarly, graphite (G) has high electronic conductivity, Fe^2+^ derived from Fe_3_O_4_ is capable of consuming e^−^ generated by light irradiation, and both synergistically reduce the complexation of e^−^/h^+^ pairs. The G-TiO_2_ @Fe_3_O_4_/PDS photocatalytic system could effectively avoid carrier complexation [151].

MXene is made of nitride, carbon nitride, and transition metal carbide, the general formula of which is M^n+1^X^n^, where M stands for the transition metal and X stands for nitrogen and/or carbon. It has excellent electrical conductivity and environmental properties. As a result of its distinctive layered nanostructure, there is more interspace for other nanoparticles, which improves their distribution on the MXene surface. MXene acts as a substrate for metal dispersion. At the same time, it provides a significant number of single-bonded F and single-bonded OH groups. One-dimensional Co (OH) F nanorods loaded in MXene offer adequate active sites for the activation of PMS [152]. MXene could be easily transformed from bulk structures to monolayer geometries by simple exfoliation due to its anisotropic properties. In addition, the transition elements contained in MXene have multi-valence states and provide abundant e^−^ export sites. Excluding the diffusion of the Ti_3_C_2_T_x_ monolayer surface’s active site, the reactiveness of the active site may contribute to its excellent catalytic performance as well, where the planar nanostructure is likely to achieve tight anchorage of PMS, triggering an effective electron transmission to accelerate ROS formation. In one study, ultrathin Ti_3_C_2_T_x_ monolayers were synthesized, and a considerable number of active sites took on the one-dimensional Ti_3_C_2_T_x_ monolayers, where the reduced size of the layer-stacked Ti_3_C_2_T_x_ significantly exposed the active sites in the ultrathin planar nanostructure and notably improved the adsorbing capacity and charge transmission capability of the PMS molecules. Thus, the high efficiency of environmental pollutants and microorganisms can be removed, and new insights and ideas for environmental purification and treatment of infectious diseases can be provided (Figure 4i) [153]. Fe_2_CoTi_3_O_10_-MXene (FCT-M) was prepared on this basis, which achieved superior activity due to the significant synergistic effect of the three transition metals (Fe, Co, and Ti) in the nanomaterials, achieving efficient degradation of organic pollutants in wastewater [154].

## 3. Metal-Free Nanomaterials

**Figure 5:**
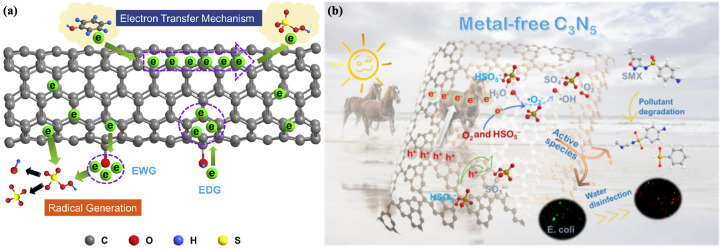
(**a**) Schematic illustration of carbon nanomaterials. Reprinted with permission from [159], Copyright 2019, Elsevier. (**b**) The use of metal−free C_3_N_5_ to activate PMS/PDS under visible light irradiation to produce SO_4_^−^·. Reprinted with permission from [167], Copyright 2021, Elsevier.

Metal-free nanomaterials are entirely made of non-metallic elements (C, N, S, P). The advantages of metal-free nanomaterials are that metal leaching does not occur in the degradation process and they are environmentally friendly. In addition, metal-free nanomaterials also have satisfactory physiochemical properties, for instance, a readily adjustable porous structure, physical and chemical stability, and acceptable cost. Accordingly, metal-free nanomaterials have been proverbially used in the activation of PDS/PMS [155]. For example, three-dimensional cubic mesoporous carbons (CMK-3) and CMK-8 serve as carbon catalysts, and the edge sites and ketone bases of the carbon materials mediate SO_4_^−^· production from peroxynitrite. CMK-3 exhibits a superior activation performance in contrast to CMK-8 due to its more stable adsorption capacity, larger superficial area, and charge-transport reduction (lesser oxygen content) [156]. One strategy to advance the catalytic efficiency of carbon nanomaterials is to carry out doping with heteroatoms in their composition, for example, doped nitrogen enhances the surface affinity and alkalinity of carbon nanomaterials, thus improving the activation capacity. For example, improvements in the performance of NCMK-3 have been demonstrated [157]. In addition to introducing elements with greater (e.g., N) or lesser (e.g., P or B) electronegativity than carbon to improve the catalytic activity, OMC (S-OMC) mixed with sulfur with a similar electronegativity to carbon introduces more defects and strains because the covalent radius of sulfur atoms is greater than carbon. In addition, the introduction of sulfur elements generates rich active sites. Surface modification and defect structure are excellent measures for enhancing the activation of nanomaterials, and the excellent electrical conductivity and affluent defect structure provide adequate active sites for activating electron generation and accelerating electron transport, respectively, for efficient application in wastewater treatment and environmental remediation [158]. It has been suggested that the chemical reactivity of carbon catalysts depends not only on structures with sawtooth edges, ketone groups, and sp^2^ carbon but also on the highly reactive carbonyl group (Figure 5a) [159]. CNTs (carbon nanotubes) consist of sp^2^-conjugated carbon atoms, the defects of which serve as significant active sites and play the part of the carrier electron transmission. The ketone group on carbon nanotubes has a couple of electrons that offer one electron to PMS to form SO_4_^−^· while it is also proposed that ND (nanodiamond) in the tetrahedral has a distinctive sp^3^ hybridization feature in the tetrahedral group and the carbonyl group on the surface of ND significantly contributes to PMS activation [160]. The use of MWCNTs (multi-walled carbon nanotubes) and GR (graphite) anodes for electrochemical activation of PMS indicates that electrolysis may increase the activation sites of carbon materials (e.g., carbonyl group), again verifying the above statement [161]. Graphite single-walled carbon nanotubes (SWCNTs) can shear the single-bond O bond of the symmetric PDS structure. Further decoration with nitrogen facilitates activation, as the nitrogen dopant synchronously induces the dipole moment with the electronegative nitrogen to transfer electrons, and electron-rich oxygen and positively charged C atoms bind atomic single-bond O bonds in peroxide. In this process, partial water oxidation occurs on the carbon surface, which acts as a transport electron channel for surface-restricted PDS or SO_4_^−^· [162]. From a large number of carbon-based nanomaterials, carbon quasi-spherical nanoparticle carbon dots (CDs) with sizes less than 10 nm stand out due to their useful properties, including upconversion properties, biocompatibility, optical stability, etc. Some studies have investigated the activation of PMS with CDs-carbon nitride photocatalysts, demonstrating the synergistic effect of CDs and carbon nitride. The synergistic effect of carbon nitride and CD has also been demonstrated by the addition of N-CDs to three kinds of carriers to form N-CDs-SiO_2_, N-CDs-CeZrO_2_, and N-CDs-Al_2_O_3_ to activate PDS [58]. Graphitic carbon nitride (g-C_3_N_4_) is a low-bandgap p-type semiconductor that is environmentally friendly, easily prepared on a large scale, and has reliable chemical and thermal stability and excellent photoelectric properties. It can be activated to form photogenic e^−^ and h^+^ by absorbing optical energy to activate PDS/PMS and generate SO_4_^−^·. Nevertheless, the rapid recombination of electron holes and the low specific area limit the activation efficiency of g-C_3_N_4_ [155]. It has been proposed that the difference in the electron density of doped atoms could form the ultrathin C_3_N_4_ nanosheet, which has high-speed charge transfer. Redistribution of the charge in O-C_3_N_4_ promoted the activation of PDS through oxygen doping while the degradation capacity of bisphenol A pollutants was greatly improved, revealing valuable insights into the exploration of high-performance catalysts for synergistic removal of environmental pollutants [163]. The photocatalyst graphene, with excellent electron mobility, was combined with g-C_3_N_4_ to form GrCN nanomaterials, which exhibited different energy band structures and restrained the recombination of the photogenerated carrier. The photothermal effect of layer-less graphite led to a significant increase in the reaction temperature, resulting in enhanced molecular collisions and conductivity of CN, which enhanced the degradation performance of pollutants [164]. Some people have designed carbon-rich defect (CN-CV_2_) structures based on the CN structure to promote the rapid capture of electrons by PDS to produce SO_4_^−^· [165]. Rhombohedral α-sulfur (α-S) and polyimide (PI), with a large specific surface area, low price, and remarkable stability, could also be activated to form photogenerated e^−^ and h^+^ by absorbing optical energy and activating PDS/PMS to produce sulfate radicals, providing a deep understanding of SR-AOPs for the removal of refractory organic pollutants and presenting better perspectives for future studies [155]. Ye et al. used deep eutectic solvent (DES) to finely regulate ramie biochar and nitrogen doping and prepared an environmentally friendly and high-performance nanomaterial for PDS activation to disinfect wastewater by destroying the cell membrane to inactivate bacteria [166]. Zhang et al. used metal-free C_3_N_5_ to activate PMS/PDS under visible light irradiation to produce SO_4_^−^·, which has a stronger PMS adsorption capacity, longer photogenerated carrier life, and higher visible light utilization efficiency, showing good emerging micro-unsustainable (Ems) degradation and water disinfection effects, which shows its role in environmental remediation and its potential for biomedicine (Figure 5b) [167].

The advantages of non-metallic nanomaterials include that it can be better adsorbed by contaminants by increasing their surface area and surface defects and there is no metal leaching. Furthermore, rejected material can be utilized as raw materials. Their disadvantages are that it is difficult to achieve the best properties due to the increased sensitivity to operating details and agglomeration may reduce the degradation efficiency [58]. Despite showing advantages regarding cost, a barrier to their widespread use is their own lower activity in contrast to metal nanomaterials. Accordingly, accurate regulation of the functional group or transformation of the surface structure is a practicable approach to enhance the activation ability and is deserving of research in the future [155].

## 4. Nanocomposites (Metal-Based Nanomaterials and Carrier)

Metal-based nanomaterials exhibit a high catalytic performance, accompanied by secondary contamination from metal leaching and an unstable performance from agglomeration, and often require binding to a carrier. Carrier materials supply attachment points for the metal to improve the electron transport efficiency and have a large surface area to adsorb contaminants such as microorganisms and better stability in wastewater and in living organisms. The coordination of the two makes use of their respective advantages reduces their shortcomings, improving the catalytic performance, and resulting in efficient SO_4_^−^· generation.

This section focuses on composites composed of metal-based nanomaterials and metal-free carriers, which usually have satisfactory chemical stability, high mechanical strength, and a large surface area. Usually, carrier nanomaterials can be classified as carbon materials (graphene, biochar, porous carbon, activated carbon, carbon spheres, etc.), silica (mesoporous silica SBA-15, fibrous silica, SiO_2_ catalytic membranes), and other non-carbon-based materials (e.g., boron nitride (BN), bacterial cellulose (BC), etc.).

**Figure 6:**
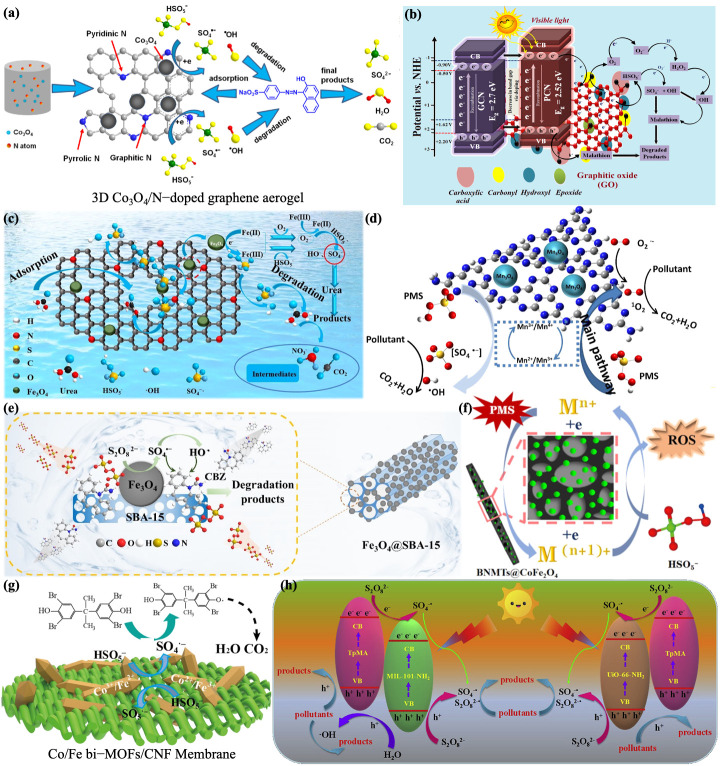
(**a**) Schematic illustration of GA loading of Co_3_O_4_. Reprinted with permission from [175], Copyright 2018, Elsevier. (**b**) Schematic illustration of the use of PCN/GO nanosheets in PMS activation for bactericidal. Reprinted with permission from [176], Copyright 2020, Springer Nature. (**c**) Schematic illustration of Fe3O4 supported on water caltrop-derived biochar for PMS activation. Reprinted with permission from [177], Copyright 2020, Elsevier. (**d**) Schematic illustration of g−C_3_N_4_. Reprinted with permission from [179], Copyright 2020, Elsevier. (**e**) Schematic illustration of SBA−15 loading of Fe_3_O_4_. Reprinted with permission from [191], Copyright 2021, Elsevier. (**f**) Schematic illustration of BN loading of CoFe_2_O_4_. Reprinted with permission from [197], Copyright 2022, Elsevier. (**g**) Schematic illustration of rod-shaped Co/Fe double MOFs, in or on CNF membranes. Reprinted with permission from [199], Copyright 2020, Elsevier. (**h**) Schematic illustration of the heterojunction structures of MOFs @COFs. Reprinted with permission from [206], Copyright 2020, Elsevier.

In accordance with the high specific surface area and conductivity of reduced graphene (rGO), magnetic NiO-NiFe_2_O_4_ [164] and Fe_3_O_4_ [168] nanoparticles have been loaded on rGO nanosheets to overcome the agglomeration problem, respectively. Additionally, to avoid potential oxidation, polydopamine (PDA) was first modified on the surface of nZVI nanoparticles and then anchored on rGO sheets to avoid the aggregation of nanomaterials. A significant adsorption ability of the contaminant was able to be realized in the case of rGO, with the π–π stacking bonds on the sheets, enhancing the degradation of the environmental pollutant phenanthrene (PHE) [165]. Some studies have scattered TiO_2_-x clusters evenly in the rGO nanosheets. Firstly, the appearance of Ti[3]^+^ improved the light-trapping ability of nanocomposites, and secondly, the nanosheets acted as an outstanding dispersion carrier to avoid aggregation of the TiO_2_ nanomaterials, which facilitated the interface reaction. Furthermore, as a result of the well-matched energy levels and close interaction between the rGO nanosheets and TiO_2_-x nanoparticles, effective separation of electron-hole pairs and fast transmission of photogenerated e^−^ were achieved, resulting in efficient degradation of micropollutants in water [169]. However, the opposite conclusion has been proposed in that the combination of rGO with TiO_2_ merely increased the quantity of •OH in contrast to pure TiO_2_ in the environment with PDS and sunlight. This decreased the decomposition pollutants’ efficiency, probably because of the reduction in the immediate photodecomposition of S_2_O_8_^2−^ and the reduction in SO_4_^−^· [170]. A previous study investigated well dispersed Zn-Co-ZIFs NPs on GO sheets, where highly active Co active sites, electron-rich ketone groups, and nitrogen doping sites contributed to the excellent catalytic activity, resulting in improved removal of sulfamethoxazole. This provides a promising heterogeneous catalyst for the elimination of refractory contaminants by SR-AOPs [171]. Hollow cobalt sulfide nanoparticles were loaded on graphene nanosheets to form Co_3_S_4_ @GN, where graphene nanosheets could restrain redundant MOF accumulation and offer nucleation sites to grow highly dispersed Co_3_S_4_ nanomaterials. This provided an adsorption domain to enable BPA to be concentrated via π–π inter-reaction, leading to the reactants forming high-concentration centers, and high-speed e^−^ transmission among the closely adsorbed BPA and SO_4_^−^· to increase the reaction rate. Being a unique radical sink, once there was a high transient concentration of SO_4_^−^·, it was confined to the microenvironment surrounding the material instead of diffusion in the contaminate solution and was applied to the catalytic degradation of bisphenol A (BPA), paving a new way for recalcitrant contaminant degradation by SR-AOPs for environmental applications [172]. Ti^3+^ and carbon quantum dot co-loaded nanocomposites (CQDs-TiO_2_-x/rGO) have been synthesized, with significantly prolonged light absorption as a result of the appearance of Ti^3+^ impurities and Ti-O-C bonds and an outstanding photon conversion performance of carbon quantum dots. rGO and CQDs with outstanding electroconductivity provide a binary channel for electron transmission, thus enabling efficacious separation of e^−^/h^+^ and fast transport of e^−^, showing great potential for environmental remediation [173]. Fe_x_Mn_y_-Fe @NCs (highly active Fe-N-C catalysts with FeN_4_ ligands) have been synthesized, with Fe-pyridine N-C acting as a sulfate-radical-generating site and FeN_4_ ligands being stable for effective iron ion dissolution [174]. The burgeoning graphene aerogels (GAs) possessed macrographically a three-dimensional monolithic structure, satisfactory electroconductivity, large specific surface area, and hierarchical porous structure, achieving multiple active sites synergistically and easy separation of loaded Co_3_O_4_ from aqueous solution (Figure 6a) [175]. Sudhaik et al. prepared PCN/GO nanosheets using phosphorus-doped graphitic carbon nitride (g-C_3_N_4_) loaded on graphene scaffolds, which facilitated charge separation and transport, increased the solar response range, and showed a rich and uniform porous structure with expanded active sites, exhibiting excellent PMS activation and thus a satisfactory bactericidal effect. This has provided inspiration for sterilization in the environment and anti-infection in the biomedical field (Figure 6b) [176].

In addition to graphene materials, other carbon-based nanomaterials have been used as negative carrier complexes. Carbon-based nanomaterials, including biochar, porous carbon, activated carbon, and carbon spheres, have been extensively researched because of their high catalytic performance, high availability, low cost, abundant folds on their surfaces, and large specific surface areas, thus providing metal-based materials with abundant binding sites for metal dispersion (Figure 6c) [177]. In addition, heteroatom doping was able to regulate the electron characteristics of primordial sp^2^ hybrid carbon, generating new active sites and increasing the specific area (e.g., doping of N) [178]. Among them, the representative graphitic carbon nitride (g-C_3_N_4_) (Figure 6d) is an ideal membrane carrier with many triangular nanopores, allowing it to permeate water and form interfacial interactions with nanoparticles through its six lone pairs of electrons in the nitrogen cavity, thus providing active sites in the reaction [179]. These carbon-based materials are shown in Table 3.

Mesoporous silica nanomaterials have attracted extensive interest in the carrier field. Among various mesoporous silica, mesoporous silica SBA-15 has a uniform porous structure, good chemical inertia, large specific area, thermal stability, biodegradability, and non-toxic chemical precursors, etc. These excellent properties mean that it can be used as a carrier material for metal oxides and an adsorbent material for microorganisms. Fe_3_O_4_ loading improved the Fe_3_O_4_ dispersion and enhanced the adsorption of PDS (Figure 6e) [191]. Using fly ash (CFA) as a silica source, the SBA-15 mesoporous molecular sieve was successfully synthesized as a carrier for MnCo_2_O_4_, which showed potential in the field of environmental remediation [192]. CMs (catalytic membranes), combining the membrane filtration process and chemical reaction, have been considered as a new strategy for a range of applications, and the membranes have exhibited an excellent separation performance and higher water fluxes with great structural flexibility to achieve sufficient reaction sites and longer contact times, thus improving the catalytic efficiency and meeting the needs of different wastewater treatments. For example, the deposition of calixarene LaFe_x_Co_1-x_O_3_-λ on SiO_2_ catalytic membranes (PCM) exhibited a high degradation performance [193]. Recently, a fiber morphology has been shown to enhance the photocatalytic degradation activity due to the highly accessible superficial area and active spots. In addition, fibers have a hole thoroughfare, and the pore size can be changed in the process of fabrication. The increased photocatalytic activity of fibrous silica titanium dioxide (FST) under visible light was mainly due to the highly accessible superficial area, crystallinity, and active spots [194]. Because SiO_2_ has a mesoporous structure and good stability, the formless SiO_2_ was coated on the outside of hollow CoS_x_ nanocages to overcome Co leaching [195]. A Co_3_O_4_/C @SiO_2_ yolk-shell nanoreactor was also designed, and the “yolk” consisted of graphitized carbon and Co_3_O_4_ nanoparticles confined in a silica shell, which also exhibited a good degradation performance toward multi-pollutants, providing a novel strategy for excellent removal of multiple pollutants in water [196].

Other non-carbon-based metal-free materials can also be used as carriers. For instance, boron nitride (BN) took on a 2D structure similar to graphite, which also had some advantages, including a large specific area, high resistance to oxidation, high thermal conductivity, numerous structural defects, and chemical durability. Its crystal structure and morphology were able to be altered to achieve a better performance, for instance, nanoribbon, nanotube, nanofiber, nanosheet, etc. The use of BN as a support skeleton for dispersing metal-based nanoparticles contributed to effectually broadening the field of application and brought new hope for activation reaction by exploiting the superiority of both, such as the effective immobilization of CoFe_2_O_4_ (Figure 6f) [197]. In addition, bacterial cellulose (BC) is a marvelous nanomaterial due to its special machinery properties, high specific area, high vertical and horizontal ratio, and uniform porous structure. BC possesses outstanding stability and membrane-forming properties, removing the recycling problem and contributing to the dispersion and accommodation of metal elements. In particular, the modified hybrid membrane treated the pollutants for at least 84 h in the flow-bed state, with a catalytic efficiency of up to 100%, and easy separation was achieved after the reaction, as demonstrated in the researcher’s study [198]. Moreover, cellulose is also an extraordinary accessible organic material in the natural world. It has a large specific area, abundant functional groups, low cost, sustainability, and biocompatibility. At the same time, CNF frequently serves as a green enhancer, where agglomeration is alleviated, and has properties such as hydrophilicity and biodegradability. The advantages of this carrier were illustrated by the uniform distribution of rod-shaped Co/Fe double MOFs in or on CNF membranes (Figure 6g) [199].

Recently, it has been published in many kinds of literature that the high loading of nanoparticles by nanofibers benefits from a large specific area. Wang et al. prepared a filter by electrostatic spinning and dispersing MOFs over the nanofibers, which showed a significant degradation efficiency for PM10 and PM2.5. Thus, their team studied ZIF-67/PAN nanofibers for the activation of PMS, exhibiting an excellent activation performance. Other researchers have also synthesized Prussian blue/PAN (PB/PAN) nanocomplexes for cesium removal. Additionally, this team also synthesized FCPBA/PAN (arachnoid Fe-Co Prussian blue analog/PAN nanofibers), which could load a significant amount of FCPBA and showed a better catalytic performance [200]. ZIF-67 was also loaded on PAN in this researcher’s study [201]. Meanwhile, PVDF (polyvinylidene fluoride) has been widely studied in distinctive membrane separation processes because of its thermal and chemical stability, high mechanical strength, flexibility (it can be made into various morphology, including blocklike, rodlike, filmlike, and porous membranes), excellent film-forming properties, and high hydrophobicity; however, the weak interface compatibility between PVDF and nanoparticles can lead to the aggregation of nanoparticles, thus hindering membrane separation. The MOF ligand made it compatible with polymers, and it was demonstrated that ZIF-67 nanoparticles were uniformly scattered over the PVDF membranes to reinforce the efficient removal of dye wastewater, demonstrating great potential for the use of self-cleaning UF membranes to treat dye wastewater [202]. In addition, functional membranes could be constructed by fibers, and carob fiber (GCF) is a natural cellulose fiber. One study compared fiber and PVDF, and composite membranes with a superior performance were identified as LDH @PVDF in terms of the reliability and separation operation difficulty [203].

Nowadays, natural minerals have widely served as the carrier of nanocatalysts because they are cost-effective, have a reusable performance, and show chemical stability. Kaolinite, a native mineral, has a 2D-layered morphology with a constant negative charge and affluent Al hydroxyl, and the 2D-layered morphology can be used as a tailored platform for grafting metal nanoparticles. Abundant Al hydroxyl and the constant negative charge prevents self-agglomeration by alleviating the crystallization of the CuFe_2_O_4_ nanoparticles on the kaolinite surface. In addition, affluent Al hydroxyl provides reactive sites to activate PMS, contributing to greater BPA degradation and thus providing an interesting insight for PMS activation using highly efficient natural mineral-based catalysts for wastewater reclamation [204]. In addition, rectorite is a typical interlayer clay mineral, and its large specific area and adsorptivity increase the relation opportunities of contaminants and nanomaterials, which may improve the removal performance. Additionally, a significant amount of •OH in rectorite plays double roles in PMS activation. On the one hand, •OH can take part in the activation of PMS. On the other hand, •OH can participate in the immobilization of metal nanoparticles, which facilitates the separation, recovery, and recycling of catalysts. Some researchers loaded FeCo_2_O_4_ on rectorite, being a steady and effective PMS activator, contributing to the practical application of sulfate-based technology for organic wastewater treatment [205]. Moreover, bentonite also serves as an excellent carrier. The anchoring of Cu&Mn-nZVFe nanoparticles via polymerization reduced the shedding and reaggregation, effectively improving the activation and performance, and showing significant potential for environmental remediation [206].

To overcome the limitation of inefficient separation and recovery from water-based media during the disinfection of wastewater, the immobilization of powder catalysts on support materials is a feasible strategy, which also prevents the agglomeration of nanomaterials, resulting in an improved activation ability. This was demonstrated in a study in which MIL-88A (Fe) nanoparticles were secured on cotton fiber to avoid secondary pollution [207]. Furthermore, 3D nanomaterials have a uniform porous morphology, which has a similar effect, resulting in the adsorption of the contaminant and e^−^ transfer during catalysis. Some researchers have synthesized sponge @MoS_2_ @GO (SMG) 3D composite nanomaterials, where the MoS_2_ nanosphere and GO were loaded over the sponge skeleton of the sandwich structure by a simple impregnation. Here, the 3D-MoS_2_ sponges, with adjustable nanostructures, interconnected pores, and hydrophobicity, enabled the recycling between Fe^2+^ and Fe^3+^ in SR-AOPs and the recovery of nanomaterials, providing better degradation of the aromatic organics and thus suggesting significant industrial applications [208]. In addition to non-metallic materials as carriers for metallic materials, both have also been designed in heterojunction structures to promote the catalytic performance. Novel MOFs @COFs (covalent organic frames) hybridized nanomaterials containing nitrogen-rich structures have been proposed as highly efficient photocatalysis platforms coupled with SR-AOPs due to their inherent surface properties of MOFs and COFs, which exhibited an excellent degradation ability for BPA. Additionally, not only was the combination of MOFs and COFs with the C_3_N_4_ active unit demonstrated to be a feasible strategy for improving the photocatalytic activities in the degradation of organic contaminants but it also provided some novel inspiration for environmental purification and biological applications (Figure 6h) [209].

## 5. Conclusions and Prospects

Although the effectiveness of nanomaterials in advanced SR-AOP has been widely demonstrated, various challenges remain to be addressed. The advantages and disadvantages of the sulfate radical-advanced oxidation of nanomaterials in biological applications are shown in Table 4. It is noteworthy that nanomaterials may gather during the preparation and activation process, and this is followed by a decrease in the surface area of the nanomaterials, resulting in a reduction in the activation efficiency [50]. On the other hand, if the catalyst contains metal nanoparticles, then metal leaching occurs, which could eventually lead to inactivation of the catalyst, secondary wastewater contamination, and biological toxicity. Hence, high chemical stability is required in order to avoid leaching of the metal components in practical applications. Usually, in order to achieve nanomaterials’ optimal activation capacity, some measures are needed, for instance, an appropriate elemental doping concentration and a specific area of nanomaterials [155]. The ability of the combination of nanomaterials and SO_4_^−^· to fight microorganisms has been tested and verified in the biological and environmental field. Nevertheless, there are arguments regarding the possible environment impact of the release of nanomaterials into the environment [50]. In many studies, a reaction intermediate/transformed product was discovered to have greater toxicity compared to the parent compound [57]. The production of toxic by-products, high levels of SO_4_^2−^, and inevitable quenching reaction are noted as the major restrictions [210]. Before practical application, the production of secondary byproducts must be taken into account. Therefore, how to limit the production of toxic by-products or control environmental hazards and cytotoxicity is worthy of further exploration [56].

As with traditional catalysts, nanocatalysts largely depend on the operating parameters, for example, the pH value, coexistence of O_2_, type of light, and number of nanomaterials used [50]. In terms of O_2_, the infectious or tumor microenvironment, which is mainly anaerobic, would affect the generation efficiency and thus affect the antibacterial and antitumor efficacy. In addition, regarding the effect of pH, the pH environment in the human body changes due to different disease conditions. Tumors and caries make the surrounding microenvironment acidic, which is conducive to the production of SO_4_^−^·. Periodontitis, on the other hand, is an alkaline environment that converts SO_4_^−^·into an inefficient •OH. These problems need to be solved [211,212].

In view of the successful implementation of SO_4_^−^· for the removal of most organic pollutants and a few antibacterial and antitumor applications, the application of SO_4_^−^· in microorganism inactivation is worthy of further exploration and research. However, the disinfection effect and inactivation mechanism of various pathogenic microorganisms have not been thoroughly studied, which is a knowledge gap. Some researchers believe that microbes are killed by the destruction of their cell membranes, cell walls, and genetic material, but this has not been well proven [31]. Therefore, systematic studies of the mechanisms of inactivation pathways and cellular sites are needed to evaluate the applicability of SO_4_^−^· in water disinfection and the biomedical field.

Although the combination of SR-AOPs with nanomaterials has been extensively studied, further research and development of innovative approaches to increase their potential is still necessary. Moreover, studies of applications in the medical field are scarce and since SO_4_^−^· indeed has many advantages over other common reactive oxygen species, this is a hot spot that should be studied. On the other hand, the mechanism of SO_4_^−^· killing of tumor cells and pathogenic microorganisms deserves more intensive investigation for better application in medicine. We look forward to the research directions and development prospects of nanomaterials involving SO_4_^−^·.

## Figures and Tables

**Table 1:** New emerging mixed-metal nanomaterials reported in the literature.

Material System	Morphological Structure	Performances	Reusability	References
CoFe_2_O_4_/PMS	Spherome, polyhedral	100% degradation of BPA in 40 min	3 recycles	[118]
CuFe-LDH/PDS/Vis	Lamellar	100% degradation of MV in 18 min	4 recycles	[119]
BiFeO_3_/PMS	Irregular crystal	94.7% degradation of bisphenol AF in 60 min	/	[120]
Cu_2_FeSnS_4_/PDS	Nano flower	98.6% degradation of BPA in 45 min	4 recycles	[121]
Fe @Fe_2_O_3_/NaHSO_3_	Linear nuclear-shell nanostructures	>99% degradation of Orange II in 30 s	/	[122]
ZnCuFe_2_O_4_/XHSO_3_/UV-vis	27 nm nanoparticle	98.6% degradation of Aatrex in 30 min	5 recycles	[123]
NiFe_2_O_4_/XHSO_3_	Graininess	87.6% degradation of estriol in 60 min	5 recycles	[124]
FeSe_2_/PMS	5–10 nm nanoparticle	82.7% degradation of PCB in 120 min, 100%degradation of PFOA in 180 min	5 recycles	[125]
LaCoO_3_/PMS	Nano stick	99.8% degradation of 2, 4-DCP in 25 min	3 recycles	[126]
La_2_CuO_4_-δ/PMS	Irregular aggregates	96% degradation of BPA in 60 min	/	[127]
Fe_x_Mo_1-x_S_2_/PDS	Nanosheet	100% degradation of aminobenzene in 20 min	5 recycles	[128]
BiVO_4_/PMS	Nanoflower-like blooming peony	100% degradation of RhB in 17 min	5 recycles	[129]
Fe-Ce/PDS	Anamorphous congeries and rod-shaped morphology	86% degradation of PAE in 6 h	/	[130]
Fe_2_O_3_/WO_3_/PMS/vis	Cluster	85.3% degradation of 4-CP in 1 h	5 recycles	[131]
Fe/TNTs/PMS	Asperous multi-layer tubular structure	95.2% degradation of APAP in 30 min	5 recycles	[132]
CeO_2_•Co_3_O_4_/PMS	Nanoflower	99% degradation of norfloxacin in 20 min	/	[133]
Co-Black TNT/PMS	Nanotubes	100% degradation of BPA and 4-CP in 15 min	10 recycles	[134]
TiO_2_ @CuFe_2_O_4_/PMS/UV	Spherical or cube	97.2% degradation of 2,4-D in 60 min	5 recycles	[135]
Fe_3_O_4_/β-FeOOH/PMS	Small cubic crystals of Fe_3_O_4_ are decorated on spindle-shaped-FeOOH	94% degradation of SMX in 30 min	/	[136]
MnO @MnO_x_/PMS	Uniform, spherical morphology of the porous structure	98.1% degradation of LEV in 30 min	3 recycles	[137]
α-Bi_2_O_3_/CuBi_2_O_4_/PMS/vis	Polyhedral, nanosphere	90% degradation of RhB in 60 min	/	[138]
DOM-FeAl-LDH/PMS	Flower-shaped	93% degradation of PBA in 60 min	/	[139]
Co-MIL-53(Al)/PMS	Cube strip	94.0% degradation of TC in 120 min	4 recycles	[140]

**Table 2:** Metal composites connected by heterojunctions reported in the literature.

Material System	Morphological Structure	Performances	Reusability	References
ZnO/Ag_6_Si_2_O_7_/PDS	The spherical particles of Ag_6_Si_2_O_7_ were located around the spindle-shaped ZnO particles	100% degradation of RhB in 150 min	5 recycles	[141]
ZnO-NiNC/PMS/vis	Dimeric particles	100% degradation of RhB in 20 min	/	[142]
Fe-POMs/TiO_2_/PDS/vis	Nanoparticles 1–2 nm in size were evenly distributed on the surface of the TiO_2_ nanosheets	100% degradation of BPA in 30 min	4 recycles	[143]
Pd/BiVO_4_/PDS/vis	The Pd nanoparticles were located on the BV nanosheets	96.43% degradation of AML in 30 min	5 recycles	[144]
Cu-Bi_2_WO_6_/PMS/LED	Cu particles were deposited on the surface of Bi_2_WO_6_ nanoparticles (porous) with damaged nanorod fragments	89.27% degradation of NOF in 60 min	5 recycles	[145]
Co-BiVO_4_/PMS	Particles of an irregular shape	99.16% degradation of BPA in 60 min	4 recycles	[146]
Bi_2_WO_6_/WO_3_/PMS	Layered construction; the WO_3_ NR is completely wrapped in ultrathin Bi_2_WO_6_ nanosheets	94.7% degradation of BPA in 30 min	3 recycles	[147]
TiO_2_/FeOCl/PDS/vis	Highly clustered near-spherical particle composition	100% degradation of RhB in 90 min	4 recycles	[148]
ZnO/CuBi_2_O_4_/PDS/vis	Sphelar CuBi_2_O_4_ particles were seen around the rice-shaped ZnO particles	100% degradation of RhB in 210 min	4 recycles	[149]

**Table 3:** The uses of carbon-based nanomaterials as carriers (except graphene) reported in the literature.

Material System	Morphological Structure	Performances	Reusability	References
Fe_3_O_4_ @BC/PMS	Layered porous structure	100% degradation of urea in 15 min	3 recycles	[177]
MnFe_2_O_4_/MS/PMS	Porous fish squamous structure, the MnFe_2_O_4_ nanoclusters on the MS surface were evenly distributed	100% degradation of orange II in 6 min	3 recycles	[180]
Fe_3_C/Fe_3_O_4_ @NC/PMS	Nuclear shell structure	87.0% degradation of CIP in 20 min	5 recycles	[181]
ZIF-NC/g-C_3_N_4_/PMS/vis	ZIF-NC: diamond-shaped dodecahedral shape, g-C_3_N_4_: lamellar	97% degradation of BPA in 10 min	5 recycles	[182]
Mn_3_O_4_-g-C_3_N_4_/PMS	Mn_3_O_4_ nanodots (5–10nm) were evenly distributed on the CNNS (g-C_3_N_4_ nanosheet)	100% degradation of 4-CP in 60 min	6 recycles	[179]
D_35_-TiO_2_/g-C_3_N_4_/PDS/vis	TiO_2_ particles with an average size of 20–52nm were distributed in the clustered, massive, and layered g-C_3_N_4_	100% degradation of BPA in 15 min	5 recycles	[183]
Co-S @NC/PMS	Nanoparticles 50–100 nm in diameter were wrapped in a graphite layer	100% degradation of DIA in 90 min	4 recycles	[184]
FON @AC/PDS/UV	FON with a spherical or cubic structure and an average size of 30 nm existed on the carbon surface	100% degradation of ACP in 60 min	5 recycles	[185]
Cu @Co-MOF/C/PMS	Small particles were dispersed on the spherical surface of Co-MOF-71; the surface of the CuCo/C structure was somewhat rough and showed a polyhedral structure	90% degradation of CIP in 30 min	4 recycles	[186]
NiFe_2_O_4_/CS/PDS	The NiFe_2_O_4_/CS particles had a larger mean diameter and a rougher sphere	67% degradation of Levofloxacin in 60 min	4 recycles	[187]
CoP @carbonand Co_3_O_4_ @carbon/PMS	Nuclear shell structure	100% degradation of phenol in 10 min	4 recycles	[188]
CuFeO @C/PMS/Vis	Irregular shape, mesoporous	89.7% degradation of LOM in 40 min	4 recycles	[189]
Ni @NCNT and SnO_2_/Ni @NCNT/PDS	Bamboo-shaped hollow shapes and delimited nanotubes, and the uniformly coated SnO_2_/Ni @NCNT exhibited an ideal porous network structure	100% degradation of CPX in 30 min	4 recycles	[190]

**Table 4:** Advantages and disadvantages of sulfate radical-advanced oxidation of nanomaterials in biological applications.

Advantages, Prospects, and Opportunities [28,29,30,32,49,50,111]	Disadvantages, Limitations, and Pitfalls [32,50,76,177,210]
SO_4_^−^· has a higher oxidation potential (2.5–3.1 V) then •OH (1.8–2.7 V).	It can cause an increase in residual cations (such as Na, K) and changes in the osmotic pressure in biology.
SO_4_^−^· has a longer lifetime (30~40 µs for SO_4_^−^· and 1 µs for •OH).	It can result in residual sulfate ions.
SO_4_^−^· has wider application conditions (such as pH) and a higher treatment efficiency.	It could cause an increase in the possibility of toxic by-products forming in the presence of Cl^−^ (such as during a saline rinse).
Nanomaterials have a specific nanostructure and larger specific area	The inevitable quenching reaction occurs when OH^−^ or Cl^−^ coexists with SO_4_^−^· (such as during a saline rinse and alkaline environments).
Nanomaterials have advantages, including high selectivity, high recoveries, and widespread optical properties.	It can convert SO_4_^−^· into an inefficient •OH in an alkaline environment (such as periodontitis).
Nanomaterials have an adjustable catalytic activity due to changes in its morphology and the application of external stimuli.	Nanomaterials gather during the preparation and activation process, resulting in a reduction in the activation efficiency
It is independent of the oxygen environment.	Metal leaching can occur, which could eventually lead to inactivation of the catalyst and biological toxicity.

## Data Availability

Not applicable.
